# Radioligand therapy of pancreatic ductal adenocarcinoma using an αvβ6-integrin targeting ^68^Ga / ^177^Lu labeled theranostic pair

**DOI:** 10.1007/s00259-025-07701-5

**Published:** 2025-12-08

**Authors:** Jan Wuestemann, Elisabeth Eppard, Daniel Hescheler, Joanna Wybranska, Dennis Kupitz, Falco Reissig, Frankis G. Almaguel, Akram Al-Ibraheem, Johannes Notni, Michael C. Kreissl

**Affiliations:** 1 Division of Nuclear Medicine, Department of Radiology and Nuclear Medicine, University Hospital Magdeburg, Leipziger Straße 44, 39120 Magdeburg, Germany; 2 TRIMT GmbH, Carl-Eschebach-Str. 7, 01454 Radeberg, Germany; 3 Department of Radiology, Wake Forest School of Medicine, Winston Salem, NC USA; 4 Department of Nuclear Medicine, King Hussein Cancer Center (KHCC), Amman, Jordan; 5 School of Medicine, University of Jordan, Amman, Jordan; 6 Institute of Pathology, School of Medicine and Health, Technical University of Munich, Trogerstr. 18, 81675 Munich, Germany

The “cancer integrin” αvβ6 is highly expressed on various carcinoma cell types [1], particularly pancreatic ductal adenocarcinoma (PDAC) [2], which has prompted the development and clinical evaluation of various αvβ6-integrin PET imaging probes [3]. However, clinical reports on corresponding therapeutic agents labelled with e.g. ^177^Lu remain scarce [4].

The image shows αvβ6-integrin targeted pretherapeutic PET/CT (A, maximum intensity projection (MIP); B, axial fusion; acquired 90 min after injection of 141 MBq of ^68^Ga-D0103) [5], as well as intratherapeutic SPECT/CT (C, E, MIP; D, F, axial fusion) of a male patient with metastatic carcinoma of the pancreatic head (60 y, 66 kg; progressive with liver metastasis, 21 months after pylorus-preserving pancreatic head resection with radical lymph node dissection, adjuvant folfirinox, gemcitabine + 5-fluorouracil, and gemcitabine + abraxane), acquired 1 day (C, D) and 3 days (E, F) after administration of the novel αvβ6-integrin targeted radioligand therapy (RLT) agent ^177^Lu-Therahexin-503 (6.925 GBq). Gelafusal^®^ (4% succinylated gelatin, 500 mL) was infused in parallel over 6 h, starting 30 min before ^177^Lu-Therahexin-503, to reduce kidney uptake [6]. The application was well tolerated without adverse effects. Complete blood count before and 48 h after therapy showed no substantial changes, all values within or close to the references. In PET/CT (A,B), a cranial (red arrow) and lateral (blue arrow) liver metastasis (MTV 2.14 and 3.14 cm^3^, respectively) showed SUVbw_max_ / SUVbw_mean_ of 12.8 / 7.7 and 13.3 / 8.5, respectively. Accordingly, a high and persisting ^177^Lu-Therahexin uptake in these lesions was confirmed by SPECT (C–F), resulting in mean absorbed doses of 9.0 ± 1.4 and 10.3 ± 3.3 Gy, respectively (voxel-based dosimetry). ^177^Lu-Therahexin SPECT/CT furthermore demonstrated negligible background and low (stomach, intestines) or moderate (liver) organ uptakes, while an initially high kidney uptake decreased over time. Taken together, ^177^Lu-Therahexin-503 showed a favourable biodistribution and a high and prolonged retention in metastatic PDAC lesions, thus holding promise for effective αvβ6-integrin targeted RLT of αvβ6-integrin expressing cancers.


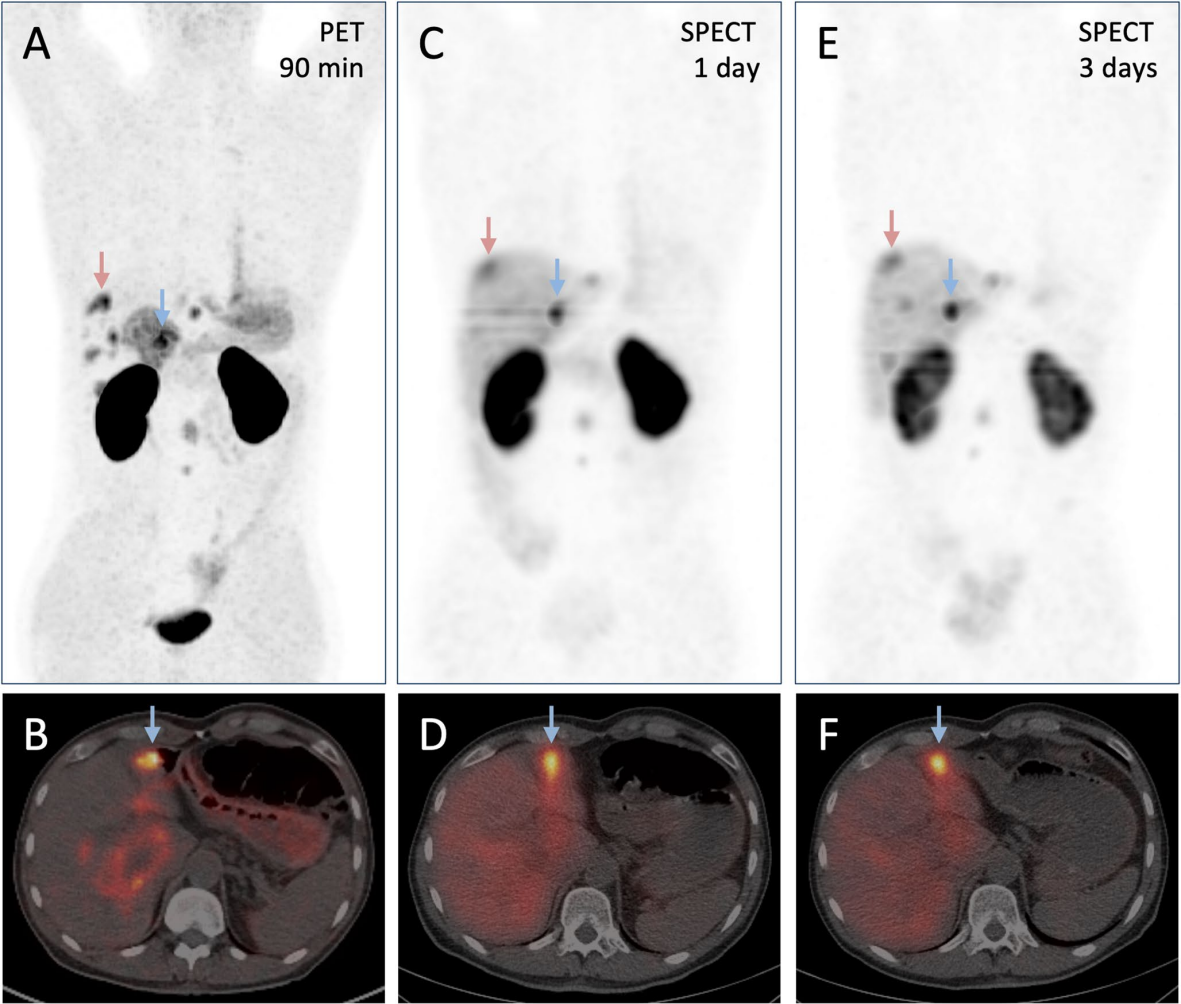


## Data Availability

The datasets used and/or analysed during the current study are available from the corresponding author on reasonable request.
